# Blinatumomab combined with low-dose chemotherapy and tyrosine kinase inhibitors as first-line induction for newly diagnosed Philadelphia chromosome-positive B-cell acute lymphoblastic leukemia: improved efficacy and reduced toxicity

**DOI:** 10.3389/fmed.2025.1687242

**Published:** 2025-11-28

**Authors:** Shujuan Xu, Xinxin Liu, Qiaoqiao Song, Guangyao Guo, Bihong Cai, Zezhong Yu, Huijian Zheng, Ping Weng, Zhenshu Xu

**Affiliations:** 1 Fujian Institute of Hematology, Fujian Provincial Key Laboratory on Hematology, Fujian Medical University Union Hospital, Fuzhou, Fujian, China; 2 Department of Hematology, The Second Affiliated Hospital of Shandong First Medical University, Tai’an, Shandong, China; 3 National Demonstration Center for Experimental General Medicine Education, Xianning Medical College, Hubei University of Science and Technology, Xianning, China

**Keywords:** Philadelphia chromosome-positive B-cell acute lymphoblastic leukemia, blinatumomab, induction therapy, low-dose chemotherapy, TKI

## Abstract

**Background:**

While blinatumomab has demonstrated substantial efficacy in relapsed/refractory B-cell acute lymphoblastic leukemia (R/R B-ALL), the therapeutic potential of combining blinatumomab with tyrosine kinase inhibitors (TKIs) and low-dose chemotherapy as frontline induction for newly diagnosed Philadelphia chromosome-positive B-ALL (Ph + B-ALL) remains systematically underexplored. This study aims to evaluate the efficacy and safety of blinatumomab in combination with low-dose chemotherapy for induction treatment of newly diagnosed Ph + B-ALL.

**Methods:**

A retrospective analysis was performed on 24 newly diagnosed cases of Ph + B-ALL admitted to the Department of Hematology at our institution, between April 2023 and April 2025.

**Result:**

The median age was 51.1 years in the blinatumomab group (*n* = 11) and 42.6 years in the conventional chemotherapy group (*n* = 13). All 24 patients attained CR after one cycle of induction chemotherapy. The MRD negativity rate showed a trend toward improvement in the blinatumomab group compared with the conventional chemotherapy group (81.8% [9/11] vs. 46.2% [6/13]). The median BCR::ABL1 level was 0.10 (range: 0.00–0.56) in the blinatumomab group versus 2.13 (range: 0.25–9.21) in the conventional chemotherapy group. The mean log reduction in the BCR::ABL1 gene level in the blinatumomab group was 3.09 ± 0.95, which was greater than in the conventional chemotherapy group (1.64 ± 0.83). At a median follow-up of approximately 13.8 months, no relapses occurred in the blinatumomab group versus 5 relapses (38.5%) in the conventional group. We documented a significant improvement in 12-month disease-free survival (DFS) rate with the blinatumomab regimen (90.9%) compared to conventional chemotherapy (46.2%). Patients receiving blinatumomab experienced markedly reduced hematologic toxicity, manifested by a shorter duration of neutropenia (2.8 ± 2.4 vs. 6.2 ± 4.4 days), a lower incidence of bleeding (0% vs. 53.8%), and a lower rate of infections (36.4% vs. 69.2%). This improved safety profile was accompanied by a significantly lower transfusion burden, as evidenced by the consumption of fewer units of platelets (1.61 ± 1.63 vs. 4.33 ± 2.90) and red blood cells (2.14 ± 2.07 vs. 6.15 ± 4.85).

**Conclusion:**

Blinatumomab combined with low-dose chemotherapy and TKIs demonstrated a trend toward improved MRD negativity, lower relapse rates, and reduced hematologic toxicity compared to conventional chemotherapy. These findings support further investigation in larger trials to confirm clinical benefit.

## 1 Introduction

Philadelphia chromosome–positive B-cell acute lymphoblastic leukemia (Ph + B-ALL) is a subtype of adult ALL with a poor prognosis that accounts for approximately 20 to 30% of newly diagnosed adult ALL (1–3). The molecular hallmark of this disease is the formation of the BCR::ABL1 fusion gene, which results in constitutive activation of tyrosine kinase activity and promotes leukemic cell proliferation (4). The introduction of TKIs, such as imatinib, combined with intensive chemotherapy has significantly improved the complete remission (CR) rate among patients with Ph + B-ALL (5–7), and hematopoietic stem cell transplantation (HSCT) following remission can also increase long-term survival outcomes (8, 9). However, the hematologic and non-hematologic toxicities associated with conventional chemotherapy regimens limit HSCT applicability in elderly patients or those with comorbid conditions. Additionally, donor shortages and transplant-related complications constrain the broader use of HSCT. As a result, disease recurrence remains high, and long-term survival rates remain suboptimal. In recent years, immunotherapy using blinatumomab, a CD19/CD3 bispecific T-cell antibody, has emerged as a novel therapeutic that can activate T-cell mediated tumor cell lysis (10, 11). The analysis of the D-ALBA study shows that a chemotherapy-free induction/consolidation regimen on the basis of blinatumomab and dasatinib is effective in inducing durable long-term hematologic and molecular responses in adult Ph + ALL (12). Currently, the clinical evidence regarding the efficacy, safety, and long-term outcomes of combining blinatumomab with TKIs and low-dose chemotherapy in newly diagnosed Ph + B-ALL patients is limited. This retrospective clinical study aimed to describe and report the clinical outcomes and safety profile observed in real-world clinical practice for this combination regimen, thereby providing evidence-based support for optimizing first-line treatment strategies.

## 2 Methods

### 2.1 Study design and patients

Twenty-four newly diagnosed patients with Ph + B-ALL were consecutively enrolled from the Department of Hematology at the Qishan Campus of Fujian Medical University Union Hospital between April 2023 and April 2025. The patients were divided into two groups based on their induction therapy: a blinatumomab combined with low-dose chemotherapy and TKI group (blinatumomab group), and a conventional chemotherapy combined with TKI group (conventional chemotherapy group). All patients fulfilled the diagnostic criteria according to morphology, immunology, cytogenetics, and molecular biology (MICM). The diagnosis of Ph + B-ALL was confirmed via chromosomal karyotyping or fluorescence *in situ* hybridization (FISH) for *t* (9;22) (q34;q11.2), and/or real-time fluorescent quantitative reverse transcription polymerase chain reaction (RQ-PCR) for the BCR::ABL1 fusion gene. Written informed consent was obtained from all patients for diagnosis and treatment.

The study collected data on patients’ age, gender, routine blood parameters, bone marrow cell morphology, immunophenotyping, cytogenetics, molecular biology markers, chemotherapy regimens, minimal residual disease (MRD), complications, survival status, and other relevant clinical information. The therapeutic efficacy was evaluated based on changes in the bone marrow profile before and after treatment. Safety was assessed by monitoring treatment-related complications. According to the “Chinese Guidelines for the Diagnosis and Treatment of Adult Acute Lymphoblastic Leukemia (2024 Edition)” (13), CR of the bone marrow is defined as a blast cell proportion of less than 5% in bone marrow samples, with no detectable blasts in peripheral blood. MRD negativity was defined as the absence of a leukemia-associated immunophenotype among ≥100,000 viable nucleated cells in bone marrow samples by multiparameter flow cytometry, corresponding to a sensitivity level of <0.01% (10^-4^). Bone marrow relapses are defined as the reappearance of blast cells (exceeding 5% in bone marrow or present in peripheral blood) or the development of extramedullary lesions following achievement of CR in patients with ALL.

### 2.2 Treatment procedure

Patients were assigned to two induction therapy groups:

Blinatumomab Group: Received reduced-intensity chemotherapy (vincristine 2 mg IV days 1, 8, 15, 22; prednisone 1 mg/kg/day days 1–14) combined with TKI. Blinatumomab was administered via continuous intravenous infusion beginning day 15 (9 μg/day days 15–20; escalated to 28 μg/day days 21–42).

Conventional Chemotherapy Group: Received the VICP regimen (vincristine 2 mg IV days 1, 8, 15, 22;idarubicin 8 mg/m^2^ IV days 1–3; cyclophosphamide 750 mg/m^2^ IV day 1; prednisone 1 mg/kg/day days 1–28) with TKI.

Following achievement of CR in both treatment groups, subsequent therapy phases (consolidation, maintenance, and central nervous system prophylaxis) were uniformly administered according to the Chinese Adult Lymphoblastic Leukemia Collaborative Group 2008 protocol (CALLG2008).

TKI therapy, consisting of either imatinib (400 mg once daily) or dasatinib (70 mg twice daily), was initiated promptly after diagnosis and maintained continuously throughout the entire chemotherapy course.

The selection of TKI and blinatumomab was guided by physician experience, patient preference, and financial considerations.

### 2.3 Adverse events

We evaluated the incidence of cytokine release syndrome (CRS) and immune effector cell-associated neurotoxicity syndrome (ICANS) according to the grading criteria proposed by Mulvey et al. (14).

### 2.4 Follow-up and observation indicators

The follow-up was censored on May 31, 2025, or at the date of patient death, whichever occurred first.

### 2.5 Statistical analysis

SPSS version 27.0 was employed for data analysis in this study. Categorical data are presented as frequencies and percentages (%), and intergroup comparisons were conducted using the chi-square test. Continuous measurement data that followed a normal distribution are expressed as mean ± standard deviation (Mean±SD), and differences between groups were analyzed using the independent samples *t*-test. For continuous data that did not conform to a normal distribution, results are reported as medians with interquartile ranges [M(P25, P75)], and group comparisons were performed using the Mann–Whitney U test. DFS, Overall Survival (OS) was estimated using the Kaplan–Meier method. It should be noted that this study was retrospective in nature and did not involve hypothesis testing; therefore, *p*-values were used solely to describe observed differences.

## 3 Result

### 3.1 Baseline patient characteristics

A total of 24 newly diagnosed Ph + B-ALL patients were enrolled in this study (Table 1). Of these, 11 were allocated to the blinatumomab plus low-dose chemotherapy and TKI regimen, while the remaining 13 received conventional chemotherapy combined with TKI. The blinatumomab group comprised 11 patients (2 males and 9 females) with a median age of 51.09 years (range, 35.0–70.0). The conventional chemotherapy group consisted of 13 patients (6 males and 7 females) with a median age of 42.62 years (range, 21.0–69.0). At baseline, the groups were comparable with respect to white blood cell count, hemoglobin level, platelet count, biochemical indices, percentage of bone marrow blasts, and BCR::ABL1 (IS) level.

**Table 1:** Baseline patient characteristics.

Patient characteristics	The blinatumomab group (*n* = 11)	The conventional chemotherapy group (*n* = 13)	Fisher/*t*/*Z* value	*p*-value
Sex			2.098	0.211
Male	2 (18.18)	6 (46.15)		
Female	9 (81.82)	7 (53.85)		
Age(y)	51.09 ± 11.92	42.62 ± 14.08	1.574	0.130
WBC (×10^9^/L)	29.53 (14.01, 95.92)	46.23 (8.84, 132.46)	−0.145	0.885
HGB (g/L)	108.64 ± 17.64	82.31 ± 26.81	−0.100	0.921
PLT (×10^9^/L)	51.82 ± 28.64	50.15 ± 54.034	0.092	0.928
Peripheral blood blasts%	41.36 ± 30.59	44.54 ± 32.21	0.221	0.827
Bone marrow blasts cells%	78.41 ± 17.00	81.23 ± 10.21	−0.246	0.808
Cytogenetics at start			2.182	0.772
Unknown	1 (9.09)	3 (23.08)		
Isolated Philadelphia chromosome	9 (81.82)	7 (53.85)		
Additional chromosomal abnormalities	1 (9.09)	3 (23.08)		
BCR–ABL1 transcript type			0.906	0.423
190	8 (72.73)	7 (53.85)		
210	3 (27.27)	6 (46.15)		
Gene quantification%	84.22 ± 27.32	88.62 ± 44.95	−0.283	0.78
Albumin (g/L)	36.18 ± 4.2	36.37 ± 7.04	−0.077	0.939
Globulin (g/L)	24.4 ± 3.47	26.46 ± 4.56	−1.228	0.233
Lactate Dehydrogenase (IU/L)	629 ± 341.5	719.54 ± 637.77	−0.422	0.677
Serum creatinine level (umol/L)	65.82 ± 16	89.77 ± 74.98	−1.036	0.311
calcium (mmol/L)	2.16 ± 0.08	2.13 ± 0.15	0.525	0.605
AST (IU/L)	45.18 ± 22.84	49.31 ± 37.76	−0.316	0.755
ALT (IU/L)	32.00 (22.00, 77.75)	37.00 (24.00, 118.50)	−0.377	0.706
TKI			−0.101	0.920
Imatinib	7 (63.64)	8 (61.54)		
Dasatinib	4 (36.36)	5 (38.46)		

### 3.2 Efficacy

All 24 patients attained CR after one cycle of induction chemotherapy. The MRD negativity rate showed a trend toward improvement in the blinatumomab group compared with the conventional chemotherapy group (81.8% [9/11] vs. 46.2% [6/13]). After one treatment course, the median BCR::ABL1 level was 0.10 (range: 0.00–0.56) in the blinatumomab group versus 2.13 (range: 0.25–9.21) in the conventional chemotherapy group. The mean log reduction in the BCR::ABL1 gene level in the blinatumomab group was 3.09 ± 0.95, which was greater than in the conventional chemotherapy group (1.64 ± 0.83). Hematological recovery, assessed by white blood cell count (WBC), neutrophil count (Neu), hemoglobin level (HGB), and platelet count (PLT), was comparable between the two groups (Table 2).

**Table 2:** Evaluation of efficacy.

Response assessment after induction therapy	The blinatumomab group (*n* = 11)	The conventional chemotherapy group (*n* = 13)	Fisher/*t*/*Z* value	*p*-value
CR			-	-
No	0 (0.00)	0 (0.00)		
Yes	11 (100.00)	13 (100.00)		
MRD-negative			1.891	0.072
No	2 (18.18)	7 (53.85)		
Yes	9 (81.82)	6 (46.15)		
Gene quantification%	0.10 (0.00, 0.56)	2.13 (0.25, 9.21)	−1.809	0.095
Log reduction in BCR-ABL1 transcript	3.09 ± 0.95	1.64 ± 0.83		0.001
WBC	4.84 ± 2.01	5.52 ± 3.54	−0.567	0.576
Neu	2.91 ± 1.37	4.21 ± 3.3	−1.392	0.183
HGB	86.55 ± 15.83	80.46 ± 13.7	1.010	0.324
PLT	206.09 ± 71.39	259.31 ± 116.43	−1.371	0.185

**Figure 1:**
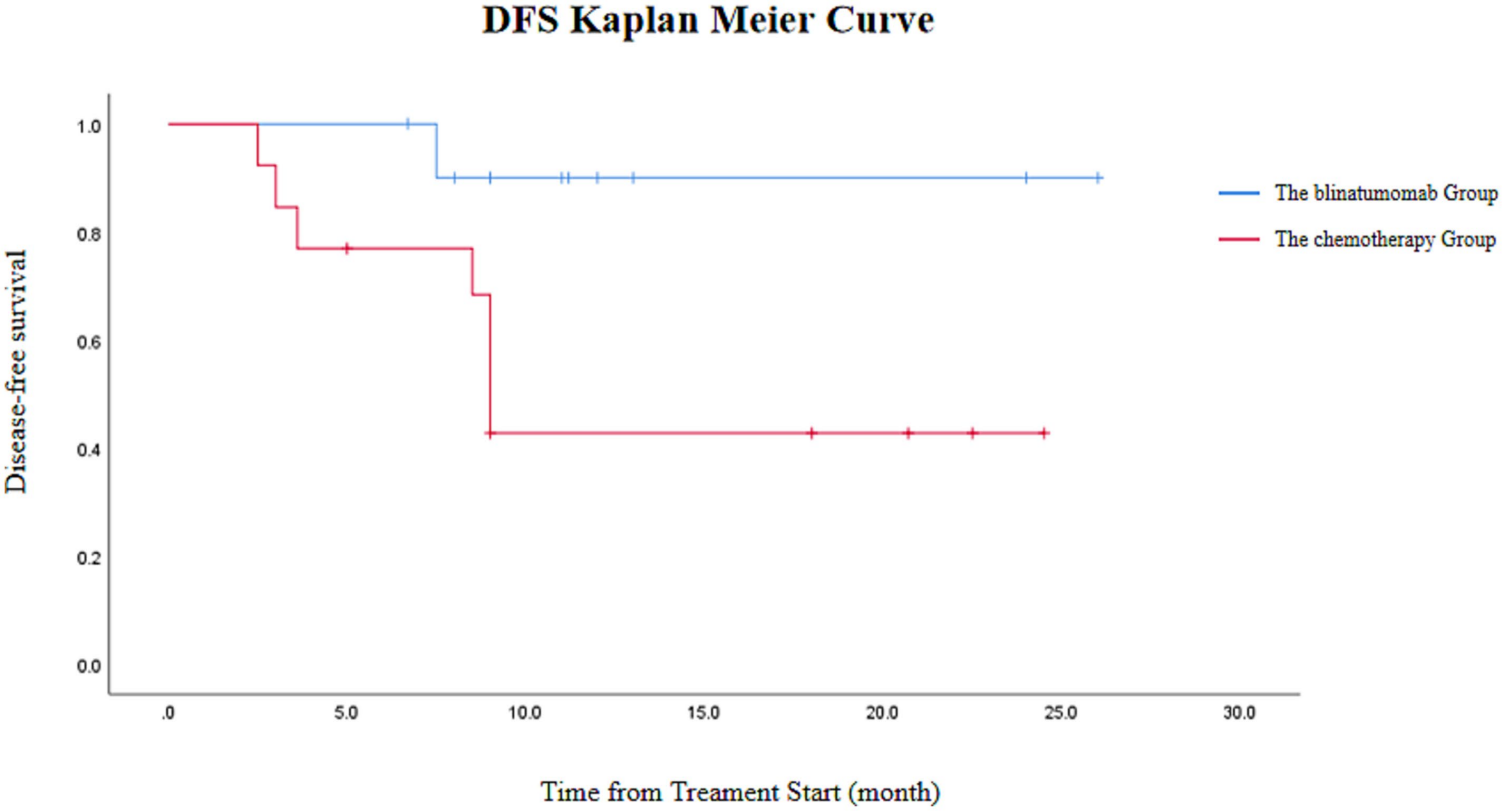
Disease-free survival (DFS) in the blinatumomab group (*n* = 11) and the conventional chemotherapy group (*n* = 13). The Kaplan–Meier curve is presented for descriptive analysis due to the limited sample size; no formal statistical comparison was performed.

At a median follow-up of 13.8 months, 4 out of 11 patients in the blinatumomab group underwent allo-HSCT post-remission, with one fatal outcome due to transplant-related complications among the transplanted patients. In the conventional chemotherapy group, 4 patients underwent allo-HSCT post-remission, with one death due to transplant-related complications and one relapse, whereas 5 of the 9 patients who continued chemotherapy experienced disease relapse. Patients in the blinatumomab group achieved significantly higher 12-month disease-free survival (DFS) rates compared with the conventional chemotherapy group (92.5% vs. 48.3%; Figure 1).

**Figure 2:**
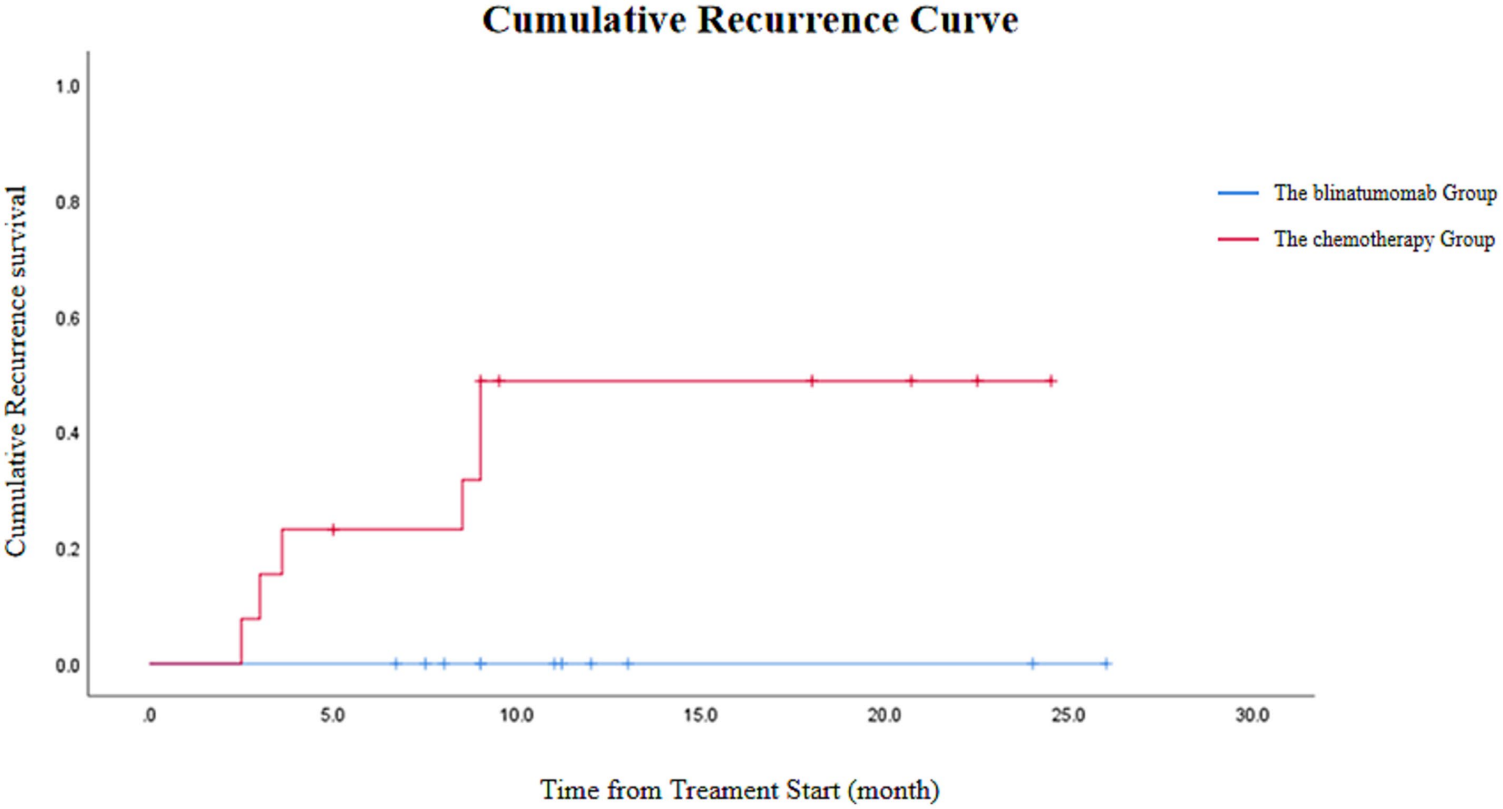
Cumulative incidence curves for relapse comparing the blinatumomab group (*n* = 11) and the conventional chemotherapy group (*n* = 13). Due to the limited sample size, this analysis is presented for descriptive purposes.

Remarkably, no relapses occurred in the blinatumomab group (0/11) during the follow-up period, compared to a relapse rate of 46.15% (6/13) in the conventional chemotherapy group (Figure 2).

**Figure 3:**
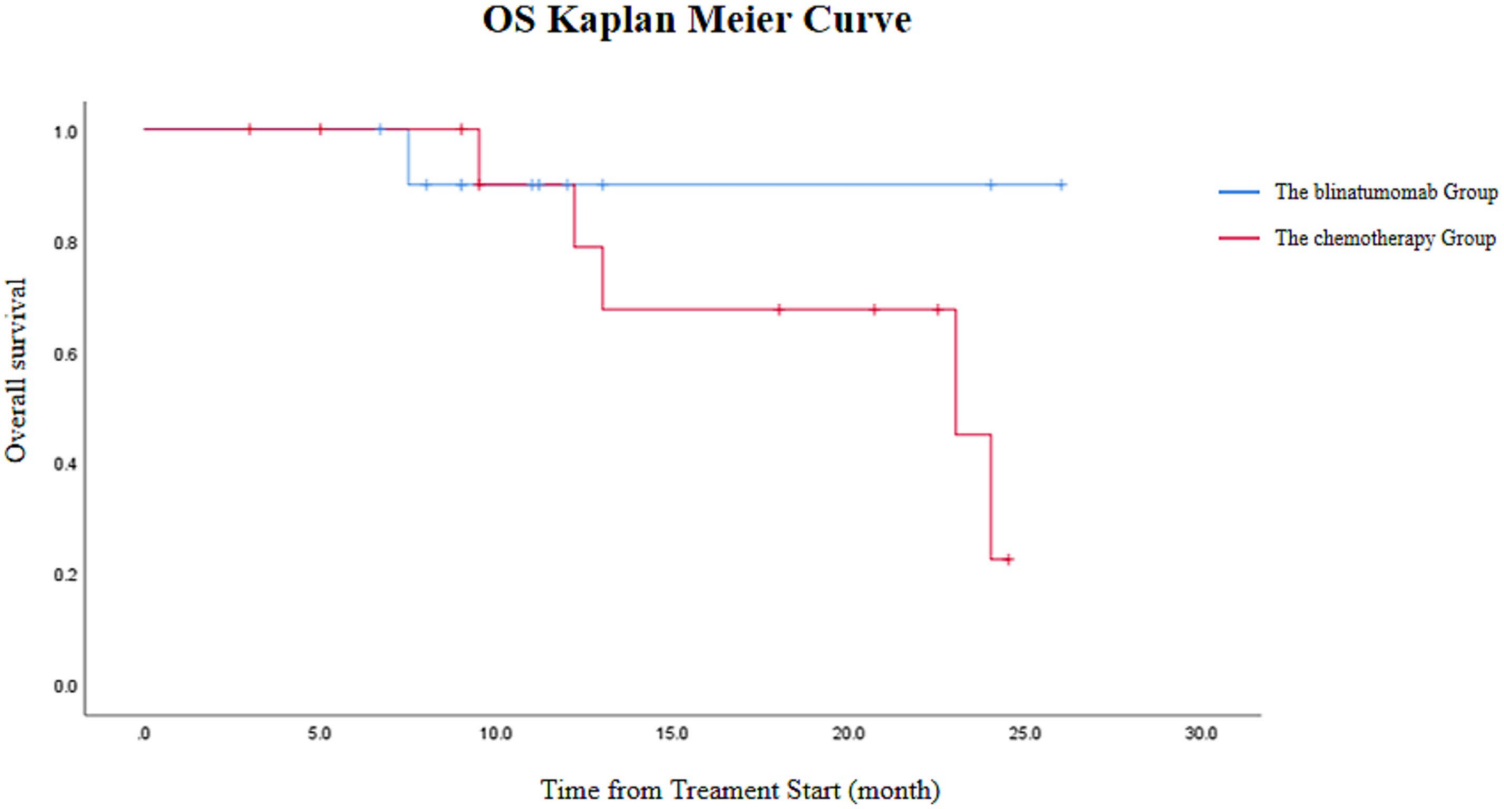
OS in the blinatumomab group (*n* = 11) versus the conventional chemotherapy group (*n* = 13). Due to the limited sample size and number of events, this Kaplan–Meier curve is presented for descriptive purposes; a formal log-rank test was not performed.

The blinatumomab group demonstrated an OS rate of 90.9%, with one death attributed to transplant-related mortality. While the conventional chemotherapy group had an OS rate of 61.5%, with four deaths from relapse and one from transplantation. The difference in OS between the two groups was not statistically significant (Figure 3).

**Figure 4:**
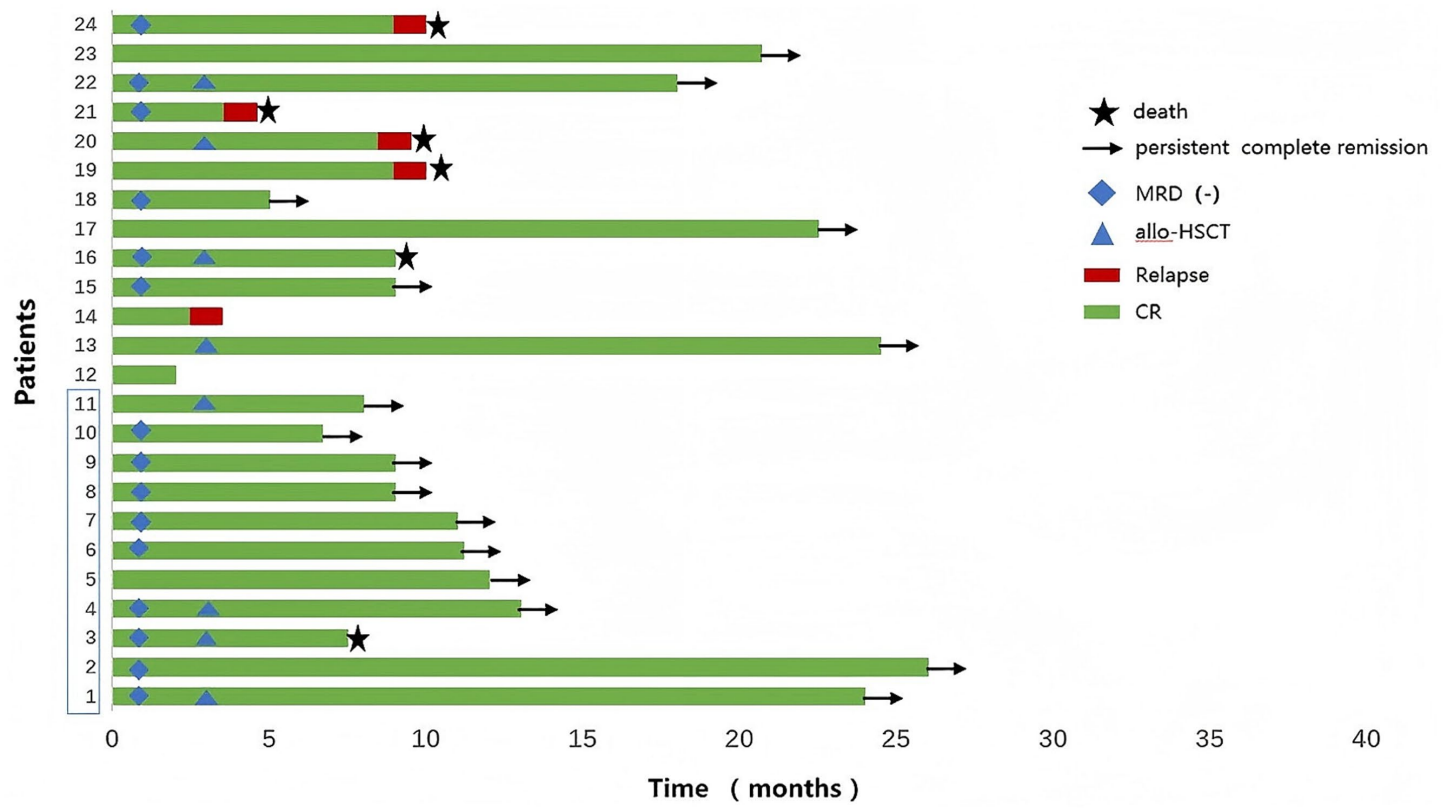
Treatment response comparison between the blinatumomab-based regimen and the conventional chemotherapy group. (Patients 1–11: blinatumomab group; Patients 12–24: conventional chemotherapy group).

Among the 24 newly diagnosed Ph + B-ALL patients, 81.82% (9/11) in the blinatumomab group achieved MRD negativity after one cycle of therapy. A high proportion of patients remained on treatment, with the longest ongoing duration exceeding 26 months. With no relapses observed, only one death occurred due to transplant-related complications. The remaining 10 patients (90.9%) continue in remission. In contrast, only 46% of patients in the conventional chemotherapy group achieved MRD negativity after one cycle, and this cohort experienced a higher rate of attrition due to disease progression. Among the 13 patients, 6 experienced relapse, 4 of whom subsequently died. Only 6 patients (46.2%) remained in continuous remission (Figure 4).

### 3.3 Safety

The incidence of bleeding events of any grade was significantly lower in the blinatumomab group compared to the conventional chemotherapy group (0% vs. 53.85%). Furthermore, the safety profile in both cohorts was devoid of any major hemorrhagic events, such as intracranial, pulmonary, or gastrointestinal bleeding. The duration of neutropenia was significantly shorter in the blinatumomab group compared to the conventional chemotherapy group (2.82 ± 2.40 days vs. 6.23 ± 4.44 days). This was corresponded to a non-significant trend toward a lower incidence of infection in the blinatumomab group (36.36% vs. 69.23%). Transfusion requirements were significantly reduced in the blinatumomab group. The mean platelet unit consumption was 1.61 ± 1.63 versus 4.33 ± 2.90, and the mean red blood cell unit usage was 2.14 ± 2.07 U versus 6.15 ± 4.85 U, both demonstrating a significant reduction compared to the conventional chemotherapy group (Table 3).

**Table 3:** Comparison of safety profiles between the blinatumomab group and the conventional chemotherapy group.

Safety profiles	The blinatumomab group (*n* = 11)	The conventional chemotherapy group (*n* = 13)	Fisher/*t*/*Z* value	*p*-value
Bleeding			8.362	0.006
No	11 (100.00)	6 (46.15)		
Yes	0 (0.00)	7 (53.85)		
Infection			2.593	0.107
No	7 (63.64)	4 (30.77)		
Yes	4 (36.36)	9 (69.23)		
Duration of Neutropenia (days)	2.82 ± 2.40	6.23 ± 4.44	−2.279	0.033
Platelet transfusion volume	1.61 ± 1.63	4.33 ± 2.90	−2.884	0.009
Blood cell transfusion (U)	2.14 ± 2.07	6.15 ± 4.85	−2.708	0.015

### 3.4 Adverse reactions

During the administration of blinatumomab, only two cases of grade 1 CRS were observed, clinically manifesting as fever. The symptoms were alleviated following symptomatic treatment with acetaminophen, and both patients tolerated the therapy well and completed the treatment course. No cases of grade 2 or higher CRS or any grade of immune effector cell-associated neurotoxicity syndrome (ICANS) were reported.

## 4 Discussion

This study retrospectively analyzed data from 24 patients with newly diagnosed Ph + B-ALL who received various induction therapies at our center. The blinatumomab-TKI combination was associated with superior efficacy and safety over traditional chemotherapy, as reflected in a higher rate of MRD-negative remission (81.8%), no relapse events, shorter neutropenia, and decreased transfusion requirements.

In the management of Ph + B-ALL traditional chemotherapy regimens have limited efficacy in achieving prolonged remission, with approximately 50% of adult patients experiencing relapses. The introduction of blinatumomab has significantly improved clinical outcomes, as patients with relapsed/refractory B-ALL and MRD-positive status have demonstrated higher molecular response rates and markedly prolonged survival (15, 16). Based on the substantial efficacy of blinatumomab observed in relapsed or refractory diseases (17, 18), researchers hypothesized that earlier administration of the drug might lead to further improvements in long-term patient outcomes. This study provides real-world evidence supporting hypothesis that the use of blinatumomab as initial induction therapy can offer significant clinical benefits to patients with Ph + B-ALL.

The MRD negativity rate in the blinatumomab group was 81.82%, compared with 46.15% in the chemotherapy group. Furthermore, the reduction in BCR::ABL1 mRNA levels was significantly greater with blinatumomab (3.09 ± 0.95 vs. 1.64 ± 0.87 log levels). The superior efficacy of this therapy arises from its novel dual-targeting mechanism, which employs a CD19 × CD3 bispecific antibody to engage endogenous T cells, thereby directing them to lyse leukemia cells (19, 20). When administered for MRD clearance following CR, blinatumomab can extend the OS of patients (21–24). In our study, it is acknowledged that BCR::ABL1 transcript levels may not have reached the threshold for molecular negativity (typically <0.01%) at the time when MRD was defined as negative by flow cytometry. This indicates divergent sensitivities and kinetics of response between flow cytometry and qPCR assays. Achieving rapid and deep remission is the critical initial step in improving the long-term prognosis for patients with Ph + ALL. Blinatumomab provides a more potent and reliable option for inducing initial remission compared to conventional chemotherapy.

The divergent response patterns observed between flow cytometric (FCM) assessment and BCR::ABL1 quantification in this study are highly instructive. In a subset of patients, FCM indicated the achievement of MRD negativity, whereas BCR::ABL1 transcripts remained persistently detectable. This phenomenon is consistent with the recent “lineage-specific clearance” theory proposed by Kim et al. (25), who demonstrated that 43% of patients harbored non-ALL derived BCR::ABL1-positive cells, a subgroup characterized by delayed BCR::ABL1 clearance. Our data, demonstrating rapid lymphoblastic clearance alongside a slower decline in BCR::ABL1 transcript levels, further corroborate this hypothesis. The biological complexity of Ph + ALL, as underscored by these findings, collectively implies that an integrated monitoring strategy—combining FCM and molecular methods—can capture complementary information. Thus, this synergy offers a more granular depiction of therapeutic responses within different cellular compartments of the disease.

No recurrence events were observed in the blinatumomab group, regardless of whether patients opted for allogeneic hematopoietic stem cell transplantation (allo-HSCT) or continued with consolidation chemotherapy following remission. In contrast, recurrence was still observed in 1/4 patients in the conventional chemotherapy group who underwent allo-HSCT after achieving remission (25%). Patients in the blinatumomab group achieved sustained remission without requiring transplant-based interventions. These findings are consistent with those reported by Short et al. (26), suggesting that effective minimal residual disease (MRD) clearance may alter the traditional transplant-dependent treatment paradigm.

The blinatumomab group exhibited a characteristic known as “selective bone marrow clearance.” The duration of neutropenia was significantly shorter at only 2.8 days, compared to 6.23 days in the conventional chemotherapy group. The requirement for platelet transfusions decreased by 67%, with an average of 1.61 doses versus 4.33 doses in the control group. Similarly, the need for red blood cell transfusions was reduced, from an average of 6.15 units in the conventional group to 2.14 units in the blinatumomab group. These reductions in transfusion requirements contribute to improved patient quality of life, lower risks associated with transfusions, such as iron overload, infection, and allergic reactions, and help alleviate the strain on medical resources. The shortened neutropenic period is the primary factor underlying the decreased incidence of infections. Notably, the infection rate in the blinatumomab group was only 36.36%, compared to 69.23% in the conventional chemotherapy group, which aligns with findings from previous studies (27, 28). Although the difference in the incidence of infection did not reach statistical significance in this study, this finding may be attributed to the limited sample size. Future studies with larger cohorts are warranted to further investigate this trend. Mechanistic research has confirmed that CD19-negative hematopoietic stem cells remain unaffected, thereby preventing chemotherapy-induced DNA damage-related hematopoietic failure (29, 30). This finding holds significant clinical value for elderly patients (the oldest participant in this study was 74 years old) and those with underlying comorbidities.

In the blinatumomab group only 18.2% experienced grade 1 CRS, which required symptomatic management alone, and no cases of grade 2 or higher CRS were observed. This outcome demonstrates improvement compared to historical data, where the CRS incidence rate was reported at 35% (31). Prior to the administration of blinatumomab a reduced-dose chemotherapy regimen was employed to decrease tumor burden. Binatumomab was then introduced using a dose-escalation strategy (9 → 28 μg/day), in combination with prednisone. The objective was to mitigate the risk of both CRS and ICANS. A domestic multicenter retrospective real-world cohort study had evaluated 20 newly diagnosed patients with B-cell acute lymphoblastic leukemia (B-ALL) who received this sequential treatment approach (32). Upon completion of induction therapy, a complete response (CR) rate of 100% and a minimal residual disease (MRD)-negative rate of 85% were achieved. Notably, no cases of ICANS or grade ≥3 CRS were reported. This therapeutic strategy successfully achieved a balanced integration of chemotherapy and immunotherapy, reducing chemotherapy-related toxicities while enhancing the effectiveness of induction therapy.

Among the 11 patients treated with blinatumomab the seven that did not undergo transplantation remained MRD-negative at the conclusion of follow-up. The European ALCANTARA study (11) similarly demonstrated comparable survival outcomes between MRD-negative patients following blinatumomab induction, irrespective of whether they received transplantation. These findings suggest a need to re-evaluate the conventional belief that transplantation is indispensable. The optimal combination of blinatumomab with chemotherapy remains to be further investigated.

We propose a novel therapeutic strategy involving “B + T” (Blinatumomab + TKI). Prior studies have indicated that the integration of blinatumomab with reduced-intensity chemotherapy in acute B-cell lymphoblastic leukemia offers certain advantages, including improved rates of CR and MRD negativity, without significantly increasing the incidence of adverse events (17). In contrast, the SWOG 1318 clinical trial reported that the induction remission rate of blinatumomab monotherapy among newly diagnosed Ph + B-ALL patients was only 66% (33), which was lower than that achieved with combination chemotherapy. The ALL08 clinical trial administered blinatumomab in conjunction with chemotherapy; however, the adverse reactions were not adequately managed due to the failure to reduce the chemotherapy dosage (34). Therefore, based on current research findings and our observational data, the combination of blinatumomab with reduced-dose chemotherapy is anticipated to emerge as a more favorable induction treatment strategy for patients newly diagnosed with Ph + B-ALL, effectively balancing therapeutic efficacy and safety.

Despite the promising findings, this study has several limitations that must be acknowledged. The limited cohort size and retrospective nature underpowered the study, which not only affected the interpretation of non-significant endpoints but also precluded robust subgroup analyses (CNA analysis, identification of patients with high-risk genetic lesions, etc). The current follow-up duration of 13.8 months remains insufficient for a conclusive assessment of long-term survival and remission durability. Therefore, our results should be considered exploratory and hypothesis-generating, setting the stage for validation in larger, prospective trials.

## 5 Conclusion

In summary, blinatumomab has demonstrated efficacy and safety as an initial induction therapy for Ph + B-ALL. It enables faster and deeper remission, with 81.82% of patients achieving MRD negativity and 100% remaining recurrence-free during the observation period. Additionally, it contributes to bone marrow preservation, effective infection control, and an improved quality of life. Furthermore, it reduces reliance on transplantation, making it suitable for elderly patients and facilitating more efficient utilization of healthcare resources.

## Data Availability

The original contributions presented in the study are included in the article/supplementary material, further inquiries can be directed to the corresponding authors.
